# *N*-(3-Chloro-4-fluoro­phen­yl)-2,2-diphenyl­acetamide

**DOI:** 10.1107/S1600536811036075

**Published:** 2011-09-14

**Authors:** A. S. Praveen, Jerry P. Jasinski, James A. Golen, B. Narayana, H. S. Yathirajan

**Affiliations:** a Department of Studies in Chemistry, University of Mysore, Manasagangotri, Mysore 570 006, India; b Department of Chemistry, Keene State College, 229 Main Street, Keene, NH 03435-2001, USA; c Department of Studies in Chemistry, Mangalore University, Mangalagangotri 574 199, India

## Abstract

In the title compound, C_20_H_15_ClFNO, the dihedral angles between the mean planes of the acetamide group and the chloro­fluoro-substituted benzene ring and the two phenyl rings are 10.8 (8), 81.9 (7) and 85.8 (5)°, respectively. The crystal packing is stabilized by N—H⋯O hydrogen bonds and weak C—H⋯O inter­molecular inter­actions, forming infinite chains along the *c* axis.

## Related literature

For the structural similarity of *N*-substituted 2-aryl­acetamides to the lateral chain of natural benzyl­penicillin, see: Mijin & Marinkovic (2006 ▶); Mijin *et al.* (2008 ▶). For their coordination abilities, see: Wu *et al.* (2008 ▶, 2010 ▶). For related structures, see: Davis & Healy (2010 ▶); Li *et al.* (2010 ▶); Li & Wu (2010 ▶); Wang *et al.* (2010 ▶); Xiao *et al.* (2010 ▶).
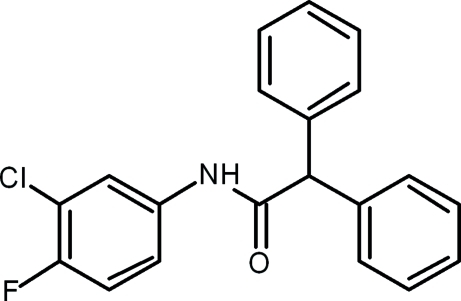


## Experimental

### 

#### Crystal data

C_20_H_15_ClFNO
*M* *_r_* = 339.78
Monoclinic, 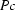

*a* = 9.3665 (17) Å
*b* = 10.2069 (12) Å
*c* = 9.7774 (16) Å
β = 114.42 (2)°
*V* = 851.1 (2) Å^3^
*Z* = 2
Mo *K*α radiation
μ = 0.24 mm^−1^
*T* = 173 K
0.35 × 0.12 × 0.12 mm

#### Data collection

Oxford Diffractio Xcalibur Eos Gemini diffractometer
Absorption correction: multi-scan (*CrysAlis RED*; Oxford Diffraction, 2010 ▶) *T*_min_ = 0.921, *T*_max_ = 0.972
7456 measured reflections
3794 independent reflections
3265 reflections with *I* > 2σ(*I*)
*R*_int_ = 0.031

#### Refinement

*R*[*F* ^2^ > 2σ(*F* ^2^)] = 0.042
*wR*(*F* ^2^) = 0.109
*S* = 1.01
3794 reflections
220 parameters
3 restraints
H atoms treated by a mixture of independent and constrained refinement
Δρ_max_ = 0.17 e Å^−3^
Δρ_min_ = −0.17 e Å^−3^
Absolute structure: Flack (1983 ▶), 1668 Friedel pairs
Flack parameter: −0.06 (6)

### 

Data collection: *CrysAlis PRO* (Oxford Diffraction, 2010 ▶); cell refinement: *CrysAlis PRO*; data reduction: *CrysAlis RED* (Oxford Diffraction, 2010 ▶); program(s) used to solve structure: *SHELXS97* (Sheldrick, 2008 ▶); program(s) used to refine structure: *SHELXL97* (Sheldrick, 2008 ▶); molecular graphics: *SHELXTL* (Sheldrick, 2008 ▶); software used to prepare material for publication: *SHELXTL*.

## Supplementary Material

Crystal structure: contains datablock(s) I. DOI: 10.1107/S1600536811036075/ya2145sup1.cif

Structure factors: contains datablock(s) I. DOI: 10.1107/S1600536811036075/ya2145Isup2.hkl

Supplementary material file. DOI: 10.1107/S1600536811036075/ya2145Isup3.cml

Additional supplementary materials:  crystallographic information; 3D view; checkCIF report

## Figures and Tables

**Table 1:** Hydrogen-bond geometry (Å, °)

*D*—H⋯*A*	*D*—H	H⋯*A*	*D*⋯*A*	*D*—H⋯*A*
C1—H1*A*⋯O1^i^	0.95	2.49	3.256 (3)	138
N1—H1*N*⋯O1^i^	0.85 (2)	2.09 (2)	2.895 (2)	158 (2)

Symmetry code: (i) 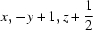.

**Fig. 1.**
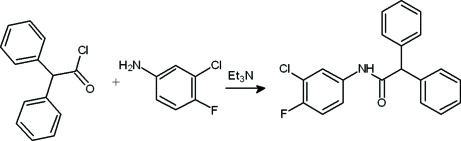
Synthesis of the title compound; reaction scheme.

**Fig. 2.**
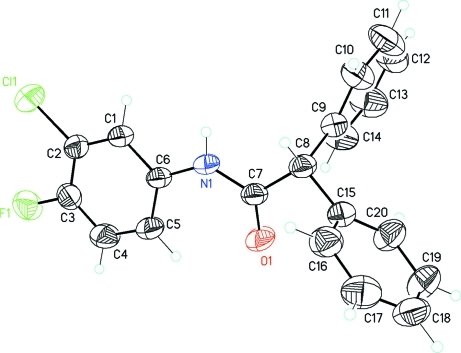
Molecular structure of the title compound; thermal displacement ellipsoids are drawn at the 50° probability level.

**Fig. 3.**
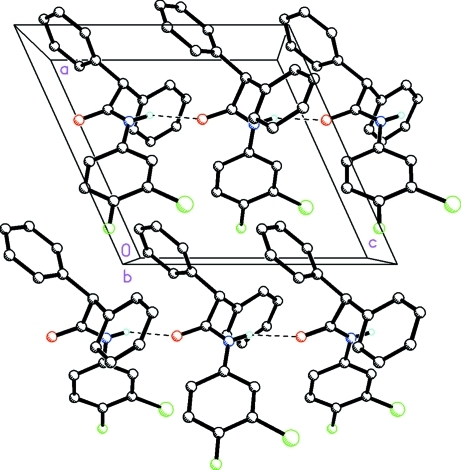
Packing diagram of the title compound viewed down the b axis. Dashed lines represent N—H···O hydrogen bonds forming infinite one-dimensional chains along the c axis; weak C-H···O interactions are not shown.

## supplementary crystallographic information

### Comment

Amides are extensively used as ligands due to their excellent coordination abilities (Wu *et al.*, 2008; 2010). N-Substituted 2-arylacetamides are especially remarkable due to their structural similarity to the lateral chain of natural benzylpenicillin (Mijin & Marinkovic, 2006; Mijin *et al.*, 2008).  Crystal structures of some acetamide derivatives, viz., 2-(4-bromophenyl)-N-(2-methoxyphenyl)acetamide (Xiao *et al.*, 2010), N-benzyl-2-(3-chloro-4-hydroxyphenyl)acetamide (Davis & Healy, 2010), 2-(2,2-dimethyl-2,3-dihydro-1-benzofuran-7-yloxy)-N- (o-tolyl)acetamide (Li *et al.*, 2010), N-benzyl-2-(2-bromophenyl)-2- (2-nitrophenoxy)acetamide (Li & Wu, 2010) and N-(4-chlorophenyl)-2-(8-quinolyloxy)acetamide monohydrate (Wang *et al.*, 2010) have been reported. In view of the importance of amides, we report herein the crystal structure of the title compound, C_20_H_15_ClFNO.

In the title compound, the dihedral angles between the mean planes of the acetamide group 01/N1/C6/C7/C8 and the chloro, fluoro substituted benzene ring C1-C6 and two phenyl rings C9-C14 and C15-C20 are 10.8 (8)°, 81.9 (7)° and 85.8 (5)°, respectively (Fig. 2).  Crystal packing is stabilized by N1—H1N···O1^i^ hydrogen bonds and weak C1—H1A···O1^i^ (symmetry code i: x, 1-y, z+1/2) intermolecular interactions forming infinite one-dimensional chains along the *c* axis (Fig. 3, Table 1).

### Experimental

Diphenylacetyl chloride (0.230 g, 1 mmol) and 3-chloro-4-fluoroaniline (0.145 g, 1 mmol) were dissolved in dichloromethane (20 mL). The mixture was stirred in the presence of triethylamine at 273 K for about 3 h (Fig. 1). The contents were poured into 100 ml of ice-cold aqueous hydrochloric acid with stirring, which was extracted three times with dichloromethane. The organic layer was washed with saturated NaHCO_3_ and brine solutions, dried and concentrated under reduced pressure to give the title compound.  Single crystals were grown from toluene by the slow evaporation method (M.P.: 427 K).

### Refinement

The N–H atom bonded to N1 was located in the diference Fourier map and refined isotropically with N-H distance constrained to 0.86 (2) Å.  All C-bound H atoms were placed in their calculated positions and included in the refinement in the riding model approximation with C–H lengths of 0.95 Å for aromatic and 1.00 Å for methyne H atoms and temperature factors set to 1.2 times *U*_eq_ of the parent atom. 1668 Friedel pairs were measured.

### Crystal data

**Table:**

C_20_H_15_ClFNO	*F*(000) = 352
*M**_r_* = 339.78	*D*_x_ = 1.326 Mg m^−^^3^
Monoclinic, *P**c*	Mo *K*α radiation, λ = 0.71073 Å
Hall symbol: P -2yc	Cell parameters from 3667 reflections
*a* = 9.3665 (17) Å	θ = 3.0–32.4°
*b* = 10.2069 (12) Å	µ = 0.24 mm^−^^1^
*c* = 9.7774 (16) Å	*T* = 173 K
β = 114.42 (2)°	Block, colorless
*V* = 851.1 (2) Å^3^	0.35 × 0.12 × 0.12 mm
*Z* = 2	

### Data collection

**Table:**

Oxford Diffractio Xcalibur Eos Gemini diffractometer	3794 independent reflections
Radiation source: Enhance (Mo) X-ray Source	3265 reflections with *I* > 2σ(*I*)
graphite	*R*_int_ = 0.031
Detector resolution: 16.1500 pixels mm^-1^	θ_max_ = 28.3°, θ_min_ = 3.0°
ω scans	*h* = −12→12
Absorption correction: multi-scan (*CrysAlis RED*; Oxford Diffraction, 2010)	*k* = −13→13
*T*_min_ = 0.921, *T*_max_ = 0.972	*l* = −12→13
7456 measured reflections	

### Refinement

**Table:**

Refinement on *F*^2^	Secondary atom site location: difference Fourier map
Least-squares matrix: full	Hydrogen site location: inferred from neighbouring sites
*R*[*F*^2^ > 2σ(*F*^2^)] = 0.042	H atoms treated by a mixture of independent and constrained refinement
*wR*(*F*^2^) = 0.109	*w* = 1/[σ^2^(*F*_o_^2^) + (0.0597*P*)^2^] where *P* = (*F*_o_^2^ + 2*F*_c_^2^)/3
*S* = 1.01	(Δ/σ)_max_ < 0.001
3794 reflections	Δρ_max_ = 0.17 e Å^−^^3^
220 parameters	Δρ_min_ = −0.17 e Å^−^^3^
3 restraints	Absolute structure: Flack (1983), 1668 Friedel pairs
Primary atom site location: structure-invariant direct methods	Flack parameter: −0.06 (6)

### Special details

**Table:**

Geometry. All esds (except the esd in the dihedral angle between two l.s. planes) are estimated using the full covariance matrix. The cell esds are taken into account individually in the estimation of esds in distances, angles and torsion angles; correlations between esds in cell parameters are only used when they are defined by crystal symmetry. An approximate (isotropic) treatment of cell esds is used for estimating esds involving l.s. planes.
Refinement. Refinement of *F*^2^ against ALL reflections. The weighted *R*-factor *wR* and goodness of fit *S* are based on *F*^2^, conventional *R*-factors *R* are based on *F*, with *F* set to zero for negative *F*^2^. The threshold expression of *F*^2^ > σ(*F*^2^) is used only for calculating *R*-factors(gt) *etc*. and is not relevant to the choice of reflections for refinement. *R*-factors based on *F*^2^ are statistically about twice as large as those based on *F*, and *R*- factors based on ALL data will be even larger.

### Fractional atomic coordinates and isotropic or equivalent isotropic displacement parameters (Å^2^)

**Table:**

	*x*	*y*	*z*	*U*_iso_*/*U*_eq_	
Cl1	0.25355 (8)	0.11734 (7)	0.82272 (8)	0.0727 (2)	
F1	0.15076 (18)	0.07394 (14)	0.50254 (19)	0.0658 (4)	
O1	0.6516 (2)	0.52072 (16)	0.54006 (16)	0.0550 (4)	
N1	0.6367 (2)	0.42807 (16)	0.74325 (18)	0.0431 (4)	
H1N	0.665 (3)	0.433 (2)	0.837 (2)	0.052*	
C1	0.4496 (3)	0.28097 (19)	0.7678 (2)	0.0446 (4)	
H1A	0.4915	0.3022	0.8716	0.053*	
C2	0.3294 (3)	0.19290 (18)	0.7097 (3)	0.0454 (5)	
C3	0.2684 (2)	0.16238 (19)	0.5588 (3)	0.0461 (5)	
C4	0.3265 (3)	0.2195 (2)	0.4665 (3)	0.0543 (5)	
H4A	0.2836	0.1976	0.3628	0.065*	
C5	0.4470 (3)	0.3086 (2)	0.5227 (2)	0.0506 (5)	
H5A	0.4865	0.3489	0.4579	0.061*	
C6	0.5107 (2)	0.33973 (18)	0.6746 (2)	0.0387 (4)	
C7	0.6985 (2)	0.51195 (18)	0.6763 (2)	0.0390 (4)	
C8	0.8312 (2)	0.59755 (18)	0.7841 (2)	0.0393 (4)	
H8A	0.8897	0.5446	0.8765	0.047*	
C9	0.7663 (3)	0.71731 (18)	0.8311 (2)	0.0433 (4)	
C10	0.8448 (3)	0.7672 (2)	0.9741 (3)	0.0582 (6)	
H10A	0.9365	0.7245	1.0431	0.070*	
C11	0.7909 (4)	0.8791 (3)	1.0177 (4)	0.0751 (8)	
H11A	0.8460	0.9124	1.1165	0.090*	
C12	0.6600 (4)	0.9418 (3)	0.9208 (4)	0.0796 (9)	
H12A	0.6245	1.0189	0.9515	0.096*	
C13	0.5799 (4)	0.8935 (3)	0.7795 (4)	0.0767 (8)	
H13A	0.4882	0.9370	0.7117	0.092*	
C14	0.6317 (3)	0.7811 (3)	0.7341 (3)	0.0628 (6)	
H14A	0.5745	0.7476	0.6357	0.075*	
C15	0.9445 (2)	0.62793 (18)	0.7125 (2)	0.0416 (4)	
C16	1.0589 (3)	0.5378 (2)	0.7245 (3)	0.0532 (5)	
H16A	1.0688	0.4600	0.7810	0.064*	
C17	1.1594 (3)	0.5589 (3)	0.6554 (3)	0.0649 (6)	
H17A	1.2372	0.4956	0.6645	0.078*	
C18	1.1470 (3)	0.6703 (3)	0.5742 (3)	0.0662 (7)	
H18A	1.2167	0.6854	0.5277	0.079*	
C19	1.0328 (3)	0.7606 (3)	0.5601 (3)	0.0662 (7)	
H19A	1.0229	0.8377	0.5024	0.079*	
C20	0.9324 (3)	0.7403 (2)	0.6290 (3)	0.0570 (6)	
H20A	0.8545	0.8038	0.6191	0.068*	

### Atomic displacement parameters (Å^2^)

**Table:**

	*U*^11^	*U*^22^	*U*^33^	*U*^12^	*U*^13^	*U*^23^
Cl1	0.0985 (5)	0.0717 (4)	0.0713 (4)	−0.0219 (4)	0.0586 (4)	−0.0035 (3)
F1	0.0645 (8)	0.0659 (8)	0.0738 (10)	−0.0204 (7)	0.0356 (7)	−0.0198 (7)
O1	0.0727 (10)	0.0649 (9)	0.0269 (7)	−0.0149 (8)	0.0200 (7)	0.0038 (6)
N1	0.0602 (10)	0.0470 (8)	0.0244 (7)	−0.0089 (8)	0.0197 (8)	−0.0019 (7)
C1	0.0650 (12)	0.0413 (9)	0.0328 (10)	−0.0007 (9)	0.0257 (9)	0.0003 (7)
C2	0.0594 (12)	0.0392 (9)	0.0494 (12)	0.0018 (9)	0.0342 (10)	0.0011 (8)
C3	0.0482 (11)	0.0405 (9)	0.0520 (13)	−0.0027 (8)	0.0232 (10)	−0.0059 (9)
C4	0.0603 (13)	0.0646 (13)	0.0345 (10)	−0.0093 (10)	0.0161 (10)	−0.0079 (9)
C5	0.0651 (13)	0.0580 (11)	0.0328 (10)	−0.0110 (10)	0.0242 (10)	−0.0025 (9)
C6	0.0514 (10)	0.0391 (8)	0.0298 (9)	0.0015 (8)	0.0208 (8)	0.0004 (7)
C7	0.0512 (10)	0.0387 (9)	0.0299 (9)	0.0038 (7)	0.0196 (8)	0.0012 (7)
C8	0.0477 (10)	0.0413 (9)	0.0305 (9)	0.0021 (8)	0.0177 (8)	0.0030 (7)
C9	0.0516 (10)	0.0440 (9)	0.0401 (10)	−0.0048 (9)	0.0248 (9)	−0.0031 (8)
C10	0.0589 (14)	0.0617 (13)	0.0506 (13)	−0.0039 (11)	0.0193 (11)	−0.0142 (11)
C11	0.0766 (17)	0.0792 (17)	0.0691 (19)	−0.0120 (15)	0.0299 (15)	−0.0369 (14)
C12	0.088 (2)	0.0621 (14)	0.101 (3)	0.0039 (15)	0.0507 (19)	−0.0263 (16)
C13	0.0832 (18)	0.0728 (17)	0.076 (2)	0.0251 (15)	0.0355 (16)	−0.0065 (15)
C14	0.0691 (15)	0.0664 (13)	0.0487 (13)	0.0169 (12)	0.0202 (12)	−0.0032 (11)
C15	0.0491 (10)	0.0435 (10)	0.0333 (10)	−0.0019 (8)	0.0181 (8)	0.0004 (7)
C16	0.0645 (13)	0.0484 (11)	0.0518 (13)	0.0084 (10)	0.0293 (11)	0.0037 (9)
C17	0.0640 (14)	0.0715 (15)	0.0666 (17)	0.0085 (12)	0.0344 (13)	−0.0051 (13)
C18	0.0688 (15)	0.0879 (18)	0.0563 (16)	−0.0157 (14)	0.0403 (14)	−0.0130 (13)
C19	0.0809 (17)	0.0669 (14)	0.0597 (16)	−0.0054 (13)	0.0381 (14)	0.0155 (12)
C20	0.0653 (14)	0.0548 (12)	0.0586 (15)	0.0061 (11)	0.0332 (12)	0.0135 (10)

### Geometric parameters (Å, °)

**Table:**

Cl1—C2	1.723 (2)	C10—C11	1.385 (4)
F1—C3	1.353 (3)	C10—H10A	0.9500
O1—C7	1.221 (2)	C11—C12	1.360 (5)
N1—C7	1.346 (3)	C11—H11A	0.9500
N1—C6	1.414 (3)	C12—C13	1.363 (5)
N1—H1N	0.847 (17)	C12—H12A	0.9500
C1—C2	1.367 (3)	C13—C14	1.388 (4)
C1—C6	1.396 (3)	C13—H13A	0.9500
C1—H1A	0.9500	C14—H14A	0.9500
C2—C3	1.379 (3)	C15—C16	1.380 (3)
C3—C4	1.363 (3)	C15—C20	1.385 (3)
C4—C5	1.374 (3)	C16—C17	1.384 (4)
C4—H4A	0.9500	C16—H16A	0.9500
C5—C6	1.389 (3)	C17—C18	1.364 (4)
C5—H5A	0.9500	C17—H17A	0.9500
C7—C8	1.528 (3)	C18—C19	1.375 (4)
C8—C9	1.519 (3)	C18—H18A	0.9500
C8—C15	1.526 (3)	C19—C20	1.380 (4)
C8—H8A	1.0000	C19—H19A	0.9500
C9—C10	1.380 (3)	C20—H20A	0.9500
C9—C14	1.387 (3)		
			
C7—N1—C6	128.12 (17)	C9—C10—C11	120.4 (3)
C7—N1—H1N	118.9 (18)	C9—C10—H10A	119.8
C6—N1—H1N	112.4 (18)	C11—C10—H10A	119.8
C2—C1—C6	120.06 (19)	C12—C11—C10	120.8 (3)
C2—C1—H1A	120.0	C12—C11—H11A	119.6
C6—C1—H1A	120.0	C10—C11—H11A	119.6
C1—C2—C3	119.92 (19)	C11—C12—C13	119.7 (3)
C1—C2—Cl1	120.96 (17)	C11—C12—H12A	120.1
C3—C2—Cl1	119.12 (17)	C13—C12—H12A	120.1
F1—C3—C4	119.8 (2)	C12—C13—C14	120.3 (3)
F1—C3—C2	119.5 (2)	C12—C13—H13A	119.9
C4—C3—C2	120.61 (19)	C14—C13—H13A	119.9
C3—C4—C5	120.3 (2)	C9—C14—C13	120.6 (3)
C3—C4—H4A	119.8	C9—C14—H14A	119.7
C5—C4—H4A	119.8	C13—C14—H14A	119.7
C4—C5—C6	119.8 (2)	C16—C15—C20	118.3 (2)
C4—C5—H5A	120.1	C16—C15—C8	119.25 (18)
C6—C5—H5A	120.1	C20—C15—C8	122.37 (19)
C5—C6—C1	119.23 (19)	C15—C16—C17	121.1 (2)
C5—C6—N1	123.94 (18)	C15—C16—H16A	119.5
C1—C6—N1	116.83 (17)	C17—C16—H16A	119.5
O1—C7—N1	122.95 (18)	C18—C17—C16	120.2 (2)
O1—C7—C8	122.26 (17)	C18—C17—H17A	119.9
N1—C7—C8	114.79 (17)	C16—C17—H17A	119.9
C9—C8—C15	114.64 (16)	C17—C18—C19	119.5 (3)
C9—C8—C7	110.86 (16)	C17—C18—H18A	120.2
C15—C8—C7	108.75 (16)	C19—C18—H18A	120.2
C9—C8—H8A	107.4	C18—C19—C20	120.6 (2)
C15—C8—H8A	107.4	C18—C19—H19A	119.7
C7—C8—H8A	107.4	C20—C19—H19A	119.7
C10—C9—C14	118.2 (2)	C19—C20—C15	120.4 (2)
C10—C9—C8	119.4 (2)	C19—C20—H20A	119.8
C14—C9—C8	122.4 (2)	C15—C20—H20A	119.8
			
C6—C1—C2—C3	−0.1 (3)	C15—C8—C9—C14	88.2 (3)
C6—C1—C2—Cl1	179.12 (15)	C7—C8—C9—C14	−35.4 (3)
C1—C2—C3—F1	179.20 (19)	C14—C9—C10—C11	−0.9 (4)
Cl1—C2—C3—F1	−0.1 (3)	C8—C9—C10—C11	177.7 (3)
C1—C2—C3—C4	−0.2 (3)	C9—C10—C11—C12	0.0 (5)
Cl1—C2—C3—C4	−179.44 (18)	C10—C11—C12—C13	0.6 (5)
F1—C3—C4—C5	−179.4 (2)	C11—C12—C13—C14	−0.2 (6)
C2—C3—C4—C5	−0.1 (3)	C10—C9—C14—C13	1.2 (4)
C3—C4—C5—C6	0.6 (4)	C8—C9—C14—C13	−177.3 (3)
C4—C5—C6—C1	−0.9 (3)	C12—C13—C14—C9	−0.7 (5)
C4—C5—C6—N1	178.7 (2)	C9—C8—C15—C16	152.1 (2)
C2—C1—C6—C5	0.7 (3)	C7—C8—C15—C16	−83.2 (2)
C2—C1—C6—N1	−178.94 (18)	C9—C8—C15—C20	−31.1 (3)
C7—N1—C6—C5	12.1 (3)	C7—C8—C15—C20	93.6 (2)
C7—N1—C6—C1	−168.3 (2)	C20—C15—C16—C17	0.2 (3)
C6—N1—C7—O1	−1.1 (3)	C8—C15—C16—C17	177.0 (2)
C6—N1—C7—C8	177.97 (18)	C15—C16—C17—C18	0.2 (4)
O1—C7—C8—C9	95.3 (2)	C16—C17—C18—C19	−0.8 (4)
N1—C7—C8—C9	−83.8 (2)	C17—C18—C19—C20	0.9 (4)
O1—C7—C8—C15	−31.6 (2)	C18—C19—C20—C15	−0.5 (4)
N1—C7—C8—C15	149.28 (17)	C16—C15—C20—C19	0.0 (4)
C15—C8—C9—C10	−90.3 (3)	C8—C15—C20—C19	−176.8 (2)
C7—C8—C9—C10	146.1 (2)		

### Hydrogen-bond geometry (Å, °)

**Table:**

*D*—H···*A*	*D*—H	H···*A*	*D*···*A*	*D*—H···*A*
C1—H1A···O1^i^	0.95	2.49	3.256 (3)	138.
N1—H1N···O1^i^	0.85 (2)	2.09 (2)	2.895 (2)	158 (2)

Symmetry codes: (i) *x*, −*y*+1, *z*+1/2.
